# Flattening the curves: on-off lock-down strategies for COVID-19 with an application to Brazil

**DOI:** 10.1186/s13362-020-00098-w

**Published:** 2021-01-06

**Authors:** Luís Tarrataca, Claudia Mazza Dias, Diego Barreto Haddad, Edilson Fernandes De Arruda

**Affiliations:** 1 Department of Computer Engineering, Celso Suckow da Fonseca Federal Center for Technological Education, Petrópolis, Brazil; 2 Department of Technologies and Languages Multidisciplinary Institute, Federal Rural University of Rio de Janeiro, Nova Iguaçu, Brazil; 3 Alberto Luiz Coimbra Institute-Graduate School and Research in Engineering, Federal University of Rio de Janeiro, Rio de Janeiro, Brazil; 4 Department of Decision Analytics and Risk, Southampton Business School, University of Southampton, 12 University Rd, Southampton, SO17 1BJ UK

**Keywords:** Neural network, Seir models, COVID-19, Coronavirus, Lockdown, Quarantine

## Abstract

The current COVID-19 pandemic is affecting different countries in different ways. The assortment of reporting techniques alongside other issues, such as underreporting and budgetary constraints, makes predicting the spread and lethality of the virus a challenging task. This work attempts to gain a better understanding of how COVID-19 will affect one of the least studied countries, namely Brazil. Currently, several Brazilian states are in a state of lock-down. However, there is political pressure for this type of measures to be lifted. This work considers the impact that such a termination would have on how the virus evolves locally. This was done by extending the SEIR model with an on / off strategy. Given the simplicity of SEIR we also attempted to gain more insight by developing a neural regressor. We chose to employ features that current clinical studies have pinpointed has having a connection to the lethality of COVID-19. We discuss how this data can be processed in order to obtain a robust assessment.

**Supplementary Information:**

The online version contains supplementary material available at 10.1186/s13362-020-00098-w.

## Introduction

The Coronavirus Disease 2019, whose aetiological agent is known as Severe Acute Respiratory Syndrome Coronavirus 2 (SARS-CoV-2) [1], has been dubbed COVID-19 by the World Health Organization (WHO). The virus has been spreading worldwide and was effectively classified as a pandemic by the WHO [2]. The first cases were reported to the Chinese bureau of WHO in December 2019 in Wuhan City, Hubei Province of China [3]. Given that the pandemic is still quite recent, several efforts are underway to try to predict its evolution, namely in terms of spread, infection rates, mortality, amongst other dimensions [4–8].

However, by checking interactive web-based dashboards (*e.g.*, [9]) it has become clear that, globally, reporting methods appear to differ substantially from country to country. Possible factors for such divergences may include lack of testing facilities, monetary constraints, geographical scale, under-reporting and even political unwillingness to divulge the true scale. Given the reliability issues related to the reported data, there is no consensus over the mortality rates associated to COVID-19 (*e.g.*, [10]). For example, while [11] argues that the rates are overestimated, [12] argue otherwise. Consequently, some have started to question whether the COVID-19 epidemic can be managed on the basis of daily data [13].

Understandably, most of the studies have focused on the contagion scenarios in Europe and China. To our knowledge, there appears to be a lack of COVID-19 related research focusing on south America, more specifically Brazil, home of approximately 211 million people, the world’s fifth-largest country by area and currently the world’s 8th largest economy. Brazil-specific predictions incorporating government introduced mitigation strategies were made available in [14] for the states of São Paulo and Rio de Janeiro. These represent the two largest economic units of the union and also concentrate a significant part of the population. However, the true local scale is difficult to assess. In part, this is due to under-reporting of cases owing to chronic test shortages [15]. Furthermore, the official figures only include deaths reported by hospitals. A more detailed analysis of current research is presented in Sect. 2.

The set of guiding questions behind this work can be stated as follows: it is common knowledge that political leadership in Brazil has at times conveyed contradictory messages on how best to tackle the crisis. Some argue for the necessity of mitigation measures, whilst others defend that these will result in insurmountable damage to the economy. As a result, can public trust in civil servants affect the epidemic? Given the current set of Brazilian public policies aiming at mitigation, how will this affect the local spread of COVID-19? What would be the effects of more relaxed non-pharmaceutical measures? Sect. 3 exploits these questions by proposing carefully designed quarantine strategies based on the availability of hospitalisation beds and evaluating these strategies in the long-term by means of a traditional SEIR epidemic model.

Furthermore, is it possible to predict how COVID-19 will affect Brazil based on what is happening in other countries? What features should be considered? Are there any peculiarities to Brazil? *E.g.* How is Brazil different from high-contagion scenarios such as Europe and the USA? How does the quality of the Brazilian health system affect the epidemic? Finally, given what we know so far about the underlying clinical conditions affecting mortality rate, how does Brazil fare? We attempt to provide an answer to these questions in Sect. 4 by employing publicly-available data alongside a neural regressor. The main conclusions of this work are presented in Sect. 5. A preliminary version of our findings was reported in [16].

## Related work

COVID-19’s human-to-human transmission is via droplets or by direct contact with an infected person [17]. An early estimate of the epidemic size in Wuhan, China, was presented in [18]. The forecast was based on the number of cases exported to international destinations. Several incubation periods have been cited in the literature, namely, 5.2 days [19] to 6.4 days [20]. Furthermore, estimates of the basic reproduction number $R_{0}$, a measure describing the average number of secondary cases resulting from an infected person, also vary widely. For example, the intervals $[2.24--3.58]$ and $[1.4--3.8]$ appear in [17] and [21], respectively.

Currently, there are multiple ongoing clinical trials worldwide to assess the effectiveness and safety of certain drugs such as chloroquine, arbidol, remdesivir, and favipiravir [22]. In vitro data has suggested that chloroquine inhibits virus replication [23], although clinical testing has failed to provide such a strong case so far. Also, clinical studies suggest the apparent efficacy of chloroquine phosphate in the treatment of pneumonia following COVID-19 infection [24]. However, as [25] carefully points out there is a delicate margin between a therapeutic and a toxic dose. The study reinforces the need for further trials to help validate the claims and design future guidelines.

Given the current lack of proven pharmaceutical solutions, most governments around the world have pursued public policies promoting social distancing, *e.g.:* closures of schools and universities, remote work when possible, travel restrictions, public gatherings bans, amongst other measures. Additional measures hinge on early detection and isolation, contact tracing, and the use of personal protective equipment [21]. These measures have been referred to as non-pharmaceutical interventions and a number of studies have been performed in order to assess the effectiveness of these strategies.

Perhaps some of the best known scientific reports coming out are the COVID-19 series produced by Imperial College. One of these is [4], which then projected 510,000 deaths in Great Britain and 2.2 million in the United States of America, in the case of an unmitigated epidemic. The authors also projected alternative scenarios in which these numbers would be revised down to, respectively 250,000 deaths and 1.1–1.2 million. They also draw attention to the fact that there is a lag between the introduction of mitigation and the corresponding decrease in hospitalization cases. At the time, their work also strongly emphasized that even for their most optimistic scenario, the number of sick people would far outstrip the available hospital capacity.

Subsequently, [5] presented an estimation of the number of infections and the impact of non-pharmaceutical interventions. This was done by using a semi-mechanistic Bayesian hierarchical model to attempt to infer the impact on 11 European countries. One of their key findings is that the decrease in the number of daily deaths being reported from Italy is in accordance with a significant impact from strict measures introduced weeks beforehand. The authors estimate that (i) between 7 and 43 million individuals, respectively 1.88% and 11.43% of the population, had been infected up to March 28th; and that (ii) 59,000 deaths had been averted through non-pharmaceutical interventions.

In [6] the authors analyse different mortality scenarios, from the absence of mitigation measures to policies designed to suppress transmission. They estimate: (i) 7.0 billion infections and 40 million deaths without mitigation; (ii) 4.5 billion infections and 20 million deaths with mitigation strategies focused on protecting elderly groups and preserving social distancing; (iii) that healthcare systems would be unable to cope even in the latter scenario. Consequently, the work strongly emphasizes the need for public health measures leading to a reduction in transmission rates, in order to avoid the collapse of global health systems.

A recent study proposed a fairly detailed dynamic model to describe the virus spread in China [7]. A drawback is that the model requires 12 parameters that are approximated from real-world data. We argue that such an approximation may lead to highly unreliable estimates given the poor quality and the reliability issues connected to the data made available. Regardless, their main findings, namely that $R_{0}$ quickly decreases with containment measures and that short quarantines do not suffice to stop the epidemics, hold true and do not depend on the quality of the data, see also [26].

A simpler SIR (susceptible-infected-recovered) model was applied to data from the UK and Italy [8]. The study suggests that (i) the epidemics originated at least a month before the first reported death and (ii) that two to three months of control measures would halt the epidemic. Although the former finding has been used to justify herd immunity strategies, that is hardly in keeping with the reported mortality rates worldwide. To illustrate the point, let us assume a mortality rate of 1% in the UK. Then, the reported figure of 167 deaths per million as of April 14 (*e.g.*, worldometers.info), would suggest the contagion of approximately 1.67% of the population. Hence, while the models are useful to guide decision, a holistic and exhaustive analysis is needed to avoid biased assessments.

A model-based analysis aimed at trying to predict mortality rates was described in [27]. The authors were able to produce age-stratified estimates of the infection fatality ratio. Their findings also estimated the mean duration from symptoms onset to fatality to be 17.8 days, whilst time from symptoms to discharge was calculated as 24.7 days. The overall fatality rate was estimated at 1.38%. However, older age groups were more afflicted. Fatality increased to 6.4% among individuals aged 60 or older and reached 13.4% of those aged 80 or older.

A study compiled and analyzed data from 1099 Chinese patients with confirmed diagnose of COVID-19 [28]. Patients most at risk of: (i) being admitted to an intensive care unit; (ii) requiring ventilator; or (iii) death included people aged 60 or older and also those with coexisting disorders such chronic obstructive pulmonary disease, diabetes, hypertension, coronary heart disease, cerebrovascular conditions, hepatitis B, cancer, chronic renal disease and compromised immune systems. Some authors have also attempted to correlate mortality rates to the Bacillus Calmette-Guérin (BCG) childhood vaccination against tuberculosis [29]. The authors found a significant negative correlation between the establishment of universal BCG vaccination and mortality rate.

### Lock-down release

After mitigating the first wave of the disease, the attention turns to finding efficient exit strategies [e.g., 26, 30, 31]. While it is perhaps consensual that a gradual and controlled exit strategy is to be preferred, gradual strategies require careful planning and coordination, as well as deft policy implementation. These attributes, however, may not be sufficient to avoid multiple waves that trigger cyclic *on-off* lock-down strategies [26]. Hence, a long-term analysis of multiple waves, as presented in Sect. 3, is essential; and it is particularly so when data is lacking and unreliable and policy making is uncoordinated, a scenario that renders multiple waves increasingly likely.

Some works have investigated optimal lock-down release strategies in [30, 31] under different performance measures. Whilst the objective in [30] is to speed-up lockdown relaxation within the limits of the healthcare system, [31] suggests a function to model the compromise between healthcare and economic issues. We argue that the search for an adequate compromise in such a complex and multifaceted problem is a very challenging task in itself, which also involves policy making and societal issues. But it is beyond the scope of this article.

Instead, we choose to model simple on-off policies to provide insight into the long-term management of multiple waves should such a management prove necessary. We start with simple on-off policies because they are simple and easy to understand and arguably easier to enforce. The choice is also practically motivated by our case study in Brazil, where mixed messages from the government response have been reported and the lack of compliance often meant that mild lock-down relaxations lead to uncontrolled spread of the disease. Such a reality renders the fall-back option of returning to full lock-down more likely than a conscious and coordinated strategy of gradual return to everyday activities.

For the sake of completeness we also consider gradual lock-down release strategies in Sect. 3.1, inspired by the results in [30, 31]. Similarly to the *on-off* policies, they are designed to keep the utilisation of hospital beds within prescribed limits, enforcing full lockdown whenever an upper bound is reached and starting the release upon reaching a lower limit. The release, however, is gradual and proceeds with a prescribed linear or exponential rate. The results show that gradual release strategies tend to reduce the full-lockdown intervals, but they do not prevent multiple waves.

## SEIR model with on/off strategy

Following the trend in the literature [e.g., 4, 5], we used the classical compartmental SEIR (susceptible, exposed, infected, removed) model to describe the virus spread. We argue that, given the uncertainty in the data, a simple but interpretable model can be more useful to provide insights for decision making. The proposed model considers a mean incubation period of 7 days [20] and a mean time to outcome (recovery or death) of 21 days, in line with [27]. We use SEIR instead of the simpler SIR model [8] because, in contrast to the latter, it includes the incubation period and allows us to replicate the delayed response to interventions in the system. To model the same spread, [32] employed a more detailed approach, whilst [8] made use of a simplified SIR model.

**Figure 1:**
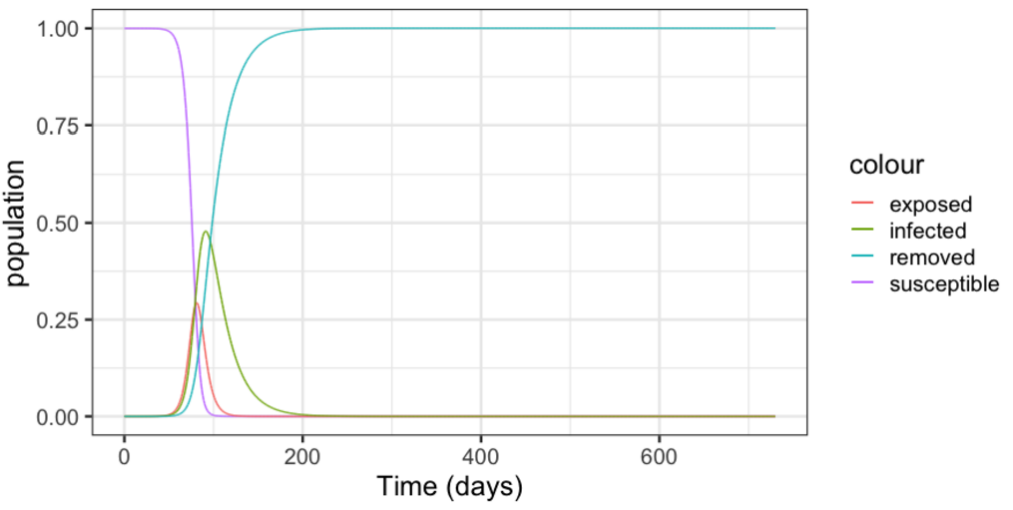
SEIR dynamics for Brazil

In order to capture the long-term behaviour, we simulated the system for a period of two years. Figure 1 depicts the dynamics. Notice the steep increase in the infected population, characteristic of the pandemic. Observe also that the proportion of infected individuals peaks around 50% of the population, which would overload any health system in the world.

The Brazilian health system has experienced a period of decreased investment and counts with around 2 hospital beds per thousand citizens [33]. To protect this system, some states in the federation are enforcing a lock-down strategy, albeit sometimes challenged by the federal government. This paper proposes a parametric on-off strategy whereby lock-down would be enforced when the number of hospitalisations due to the epidemic approaches the total number of hospital beds, and removed when the occupation recedes to a lower threshold. For the sake of simulation, we assume a hospitalisation rate of 10% [e.g., 27]. Hence, the lock-down and relaxation thresholds can be alternatively set in terms of the total number of infected patients. Our simulations do not consider the development of curative medication or of an effective vaccine in a two-year horizon. Naturally, should any of these developments occur, the control strategies would have to be completely reformulated.

**Figure 2:**
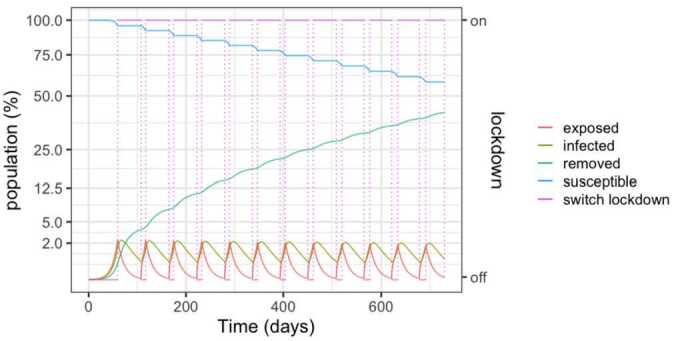
SEIR dynamics for 25% and 75% thresholds

The first strategy is reported in Fig. 2 and corresponds to activating lock-down whenever the number of infections overcomes 1.5% of the population, which corresponds to an occupation of 1.5 beds per thousand inhabitants (75% of the beds). Conversely, the lock-down is relaxed when the bed occupation reaches 25%, or infection decreases below 0.5% of the population. Notice that after two years, nearly 40% of the population will have been infected and therefore be possibly immune. When one considers the results later described in Sect. 4, this also means the death of around one to three percent of the population (2.9% to 8% of the infected population).

**Figure 3:**
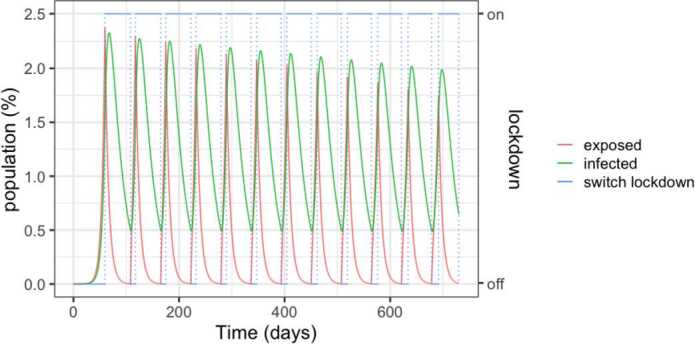
SEIR dynamics for 25% and 75% thresholds, zoom

Figure 3 details the evolution of the infected and exposed populations. Observe that, even though the control policy is set for a 1.5% threshold, the number of infected individuals exceeds 2% in the peaks because exposed individuals become infected after the onset of the lock-down. Moreover, the peaks decrease over time, as the susceptible population goes down. Notice also that the lock-down periods alternate with comparatively small relaxation intervals.

**Figure 4:**
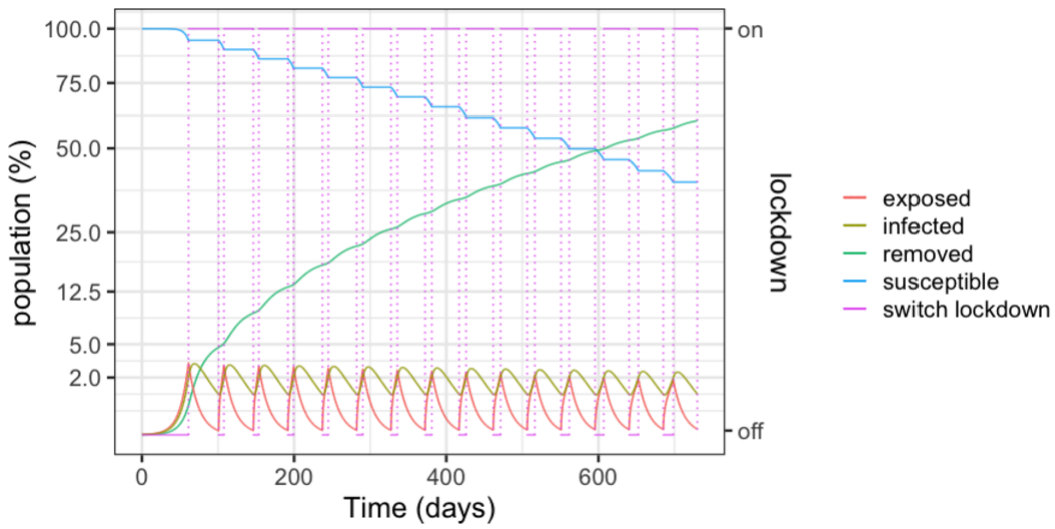
SEIR dynamics for 50% and 100% thresholds

Figure 4 depicts the populations for a 50%–100% strategy. Lock-down is enforced when hospital beds are full and relaxed when less than half are occupied. With respect to the 25%–75% policy, we observe an increase in the infected population, with about 60% of the population being infected after two years. This is due to the increased occupation in the health system. Unfortunately, in view of the results that will be presented Sect. 4, the result implies the death of 1.5% to 4.6% of the population.

**Figure 5:**
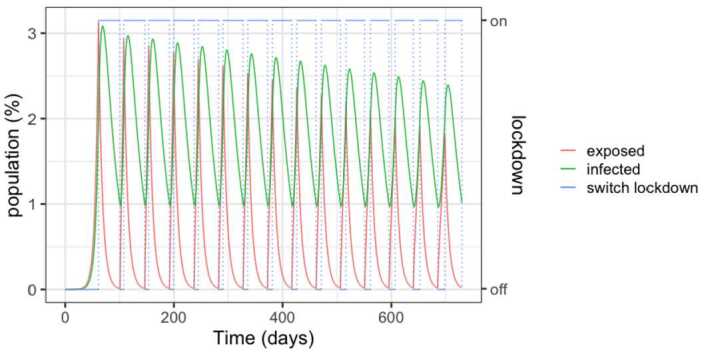
SEIR dynamics for 50% and 100% thresholds, zoom

However, as detailed in Fig. 5, the number of required beds is in excess of 3 per thousand inhabitants in the early peaks, signalling that a significant expansion of the health system would be needed. Another insight of the simulations is that the relaxations have to be carefully studied and the thresholds carefully calibrated in order to avoid the collapse of the health system.

### Gradual release strategies

We expand our analysis by introducing gradual release policies with the same upper and lower threshold parameters as the on-off strategies. The difference here is that the release is gradual and can occur at a linear or exponential rate. For the sake of simulation, the linear release rate is set to $\frac{1}{90}$, meaning that total release would be achieved within three months. While the analysis is not intended to be exhaustive, it does provide insights regarding the long-term consequences of gradual releases. Specific analysis for local realities can be implemented making use of the R code provided as Additional file 1.

**Figure 6:**
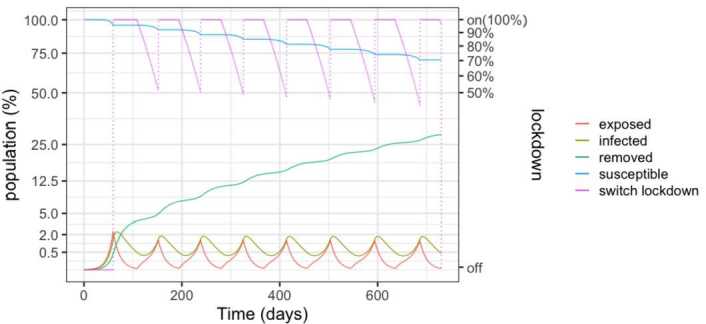
25%–75% strategy with linear release

Figure 6 depicts the dynamics resulting from the 25%–75% policy under linear release. Comparing with the corresponding on-off policy (Fig. 2), we notice an increase in the length of periods between full lockdowns. This is to be expected since a gradual release implies lower transmission levels. Observe, however, that the gradual release does not avoid subsequent peaks that enforce full lock-downs; indeed, even though the level of release before the next full lockdown increases over time, it never substantially surpasses 50%. That may be due to the reduced hospital capacity.

**Figure 7:**
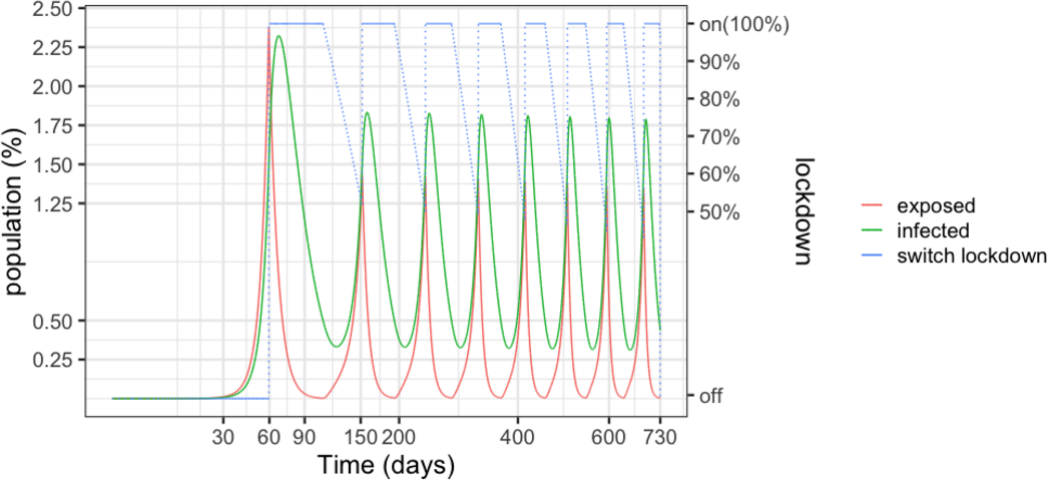
25%–75% strategy with linear release-zoom

Figure 7 shows that the gradual release improves the quality of the control, reducing the infection peaks. Because the peaks are less pronounced, a lower percentage of people will have been infected at the end of two years—around 30% as opposed to 45% for the pure on-off strategy, see Figs. 2 and 6. It is worth reinforcing that, depending on the perceived compromise between economy and healthcare, either policy could be advocated.

**Figure 8:**
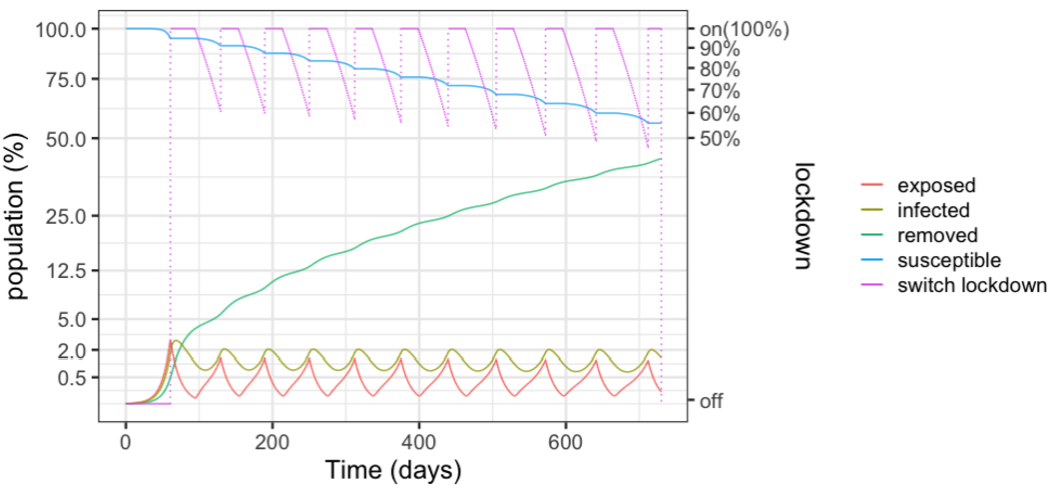
60%–90% strategy with linear release

**Figure 9:**
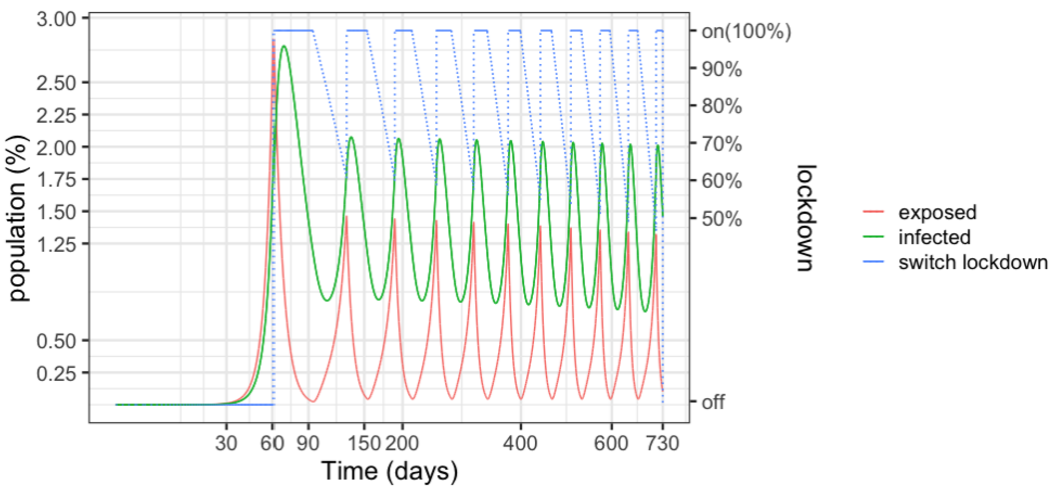
60%–90% strategy with linear release, zoom

Figures 8–9 illustrate a linear release policy for 60% and 90% thresholds. Because of the increased hospital utilisation with respect to the last example, the number of infected (recovered) individuals at the end of the two-year window approaches 50%. Moreover, we also notice a more pronounced reduction in the length of the full lockdown intervals, with similar lock-down release levels over time.

We also conducted tests with an exponential release with half life of 30 days, in which $u(t) = 1 - e^{-0.231 t - t_{0}}$ is the level of lock-down *t* units of time after the end of the last full lock-down (at $t_{0}$). The results are very similar to those of the corresponding linear release strategies and are omitted for the sake of brevity. The results seem to suggest that, regardless of the release strategies, cycles of lock-down and release may be necessary to curb the disease. Therefore, a careful analysis of such cycles is essential.

## Neural prediction of the Brazilian case fatality rate

The death toll due to COVID-19 in different scenarios is one of the most important quantities to forecast. It can be inferred from the number of infected individuals when the overall case fatality rate (CFR) is known. Unfortunately, the reported Brazilian CFR is not reliable, due in part to insufficient testing [15]. Bearing that in mind, we propose a model to predict the Brazilian CFR based on information acquired from COVID-19 data repositories worldwide.

**Figure 10:**
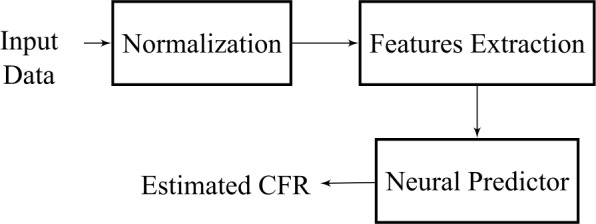
Block diagram of the architecture of a single neural predictor (*i.e.*, a single model)

**Figure 11:**
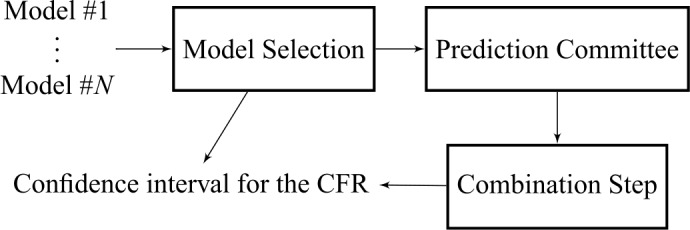
Block diagram of the proposed committee machine. Note that the combination step is the median operator, and that the confidence interval can be computed using variability statistics derived from the “Model Selection” procedure

The proposed model utilises a committee of neural predictors, each with the architecture depicted in Fig. 10. The committee is able to combine individual weak predictors in order to produce an improved overall regression [34]. Given the variation of the data, and considering the reliability issues surrounding multiple data sources, we use the median of the weak predictors to hedge against outliers [34, 35]. Figure 11 illustrates the committee strategy. This paper utilises a committee of $N=3$ distinct regressors and the median operator. Thus, the final committee estimate *y*, from a set $\{y_{1},y_{2},\ldots,y_{N}\}$ of estimates (one for each individual regressor) is computed from

1 $$ y = \operatorname{median}\{y_{1},y_{2}, \ldots,y_{N}\}. $$

Note that the CFR strongly depends on several risk factors, which can be related to either individual or societal features. Among the former, one finds chronic medical conditions (especially diabetes [36], cardio-cerebrovascular diseases [37], hypertension [38] and respiratory system diseases [1]), pregnancy [39], obesity [40] and advanced age [38]. We can argue that social factors that influence the CFR have attracted less attention, although their impact cannot be dismissed as negligible. Among these factors, one may emphasise: shortage of medical protection in developing countries [41], risk perception by the community [42], political commitment to allocate resources in order to reduce disaster risks [43], disaster risk governance [44], appropriate allocation of humanitarian response and development activity [45], participatory approaches that change risk management [46], and institutional differences [47]. It is a challenging task to incorporate such factors in a regression model, mainly due to the absence of reliable metrics for the majority of countries that have experienced COVID-19 dissemination. Fortunately, there are some quantitative features available for the countries of interest that are correlated with the aforementioned factors (*e.g.*, it is expected that the indicator “Enforcement of regulations”, provided by the Legatum Institute, is correlated with institutional differences among different countries). Overall, a total of eight features (Table 1) were selected as inputs to the neural predictors, namely [48]:

- *Obesity:* Percentage of the adult population who have obesity;
- *Smoking:* Percentage of the 15+ population who currently smoke any tobacco product on a regular basis;
- *Healthcare coverage:* Percentage of population without healthcare coverage, either through private insurance, or state-provided coverage (regardless of whether they are able to effectively access healthcare through that coverage);
- *Raised blood pressure:* Percentage of the 18+ population with raised blood pressure;
- *Public Trust in Politicians:* Expert survey (1–7 scale), of how the ethical standards of politicians are rated;
- *Regulation Enforcement:* Composite measure of whether government regulations, such as labour, environmental, public health, commercial, and consumer protection regulations, are effectively enforced; expert survey (0–1 scale);
- *Population over age 65:* Percentage of the adult population who are 65+;
- *Cardiovascular fatalities:* Percentage of the adult population whose fatalities are a result of cardiovascular diseases.

**Table 1:** Input features utilized for the CFR neural regressor. Sources: LT (Legatum Institute), WBDI (World Bank Development Indicators), WHO (World Health Organization Global Estimates 2016), ILO (International Labour Organization), WEF (World Economic Forum), WJP (World Justice Project)

Variable	Indicator	Source
$x_{1}$	Obesity	LT / WHO
$x_{2}$	Smoking	LT / WHO
$x_{3}$	Healthcare coverage	LT / ILO
$x_{4}$	Raised blood pressure	LT / WHO
$x_{5}$	Public trust in politicians	LT / WEF
$x_{6}$	Enforcement of regulations	LT / WJP
$x_{7}$	Population over age 65 (%)	WBDI
$x_{8}$	Fatalities of cardiovascular diseases (%)	WHO

Some of the features employed, namely: Obesity, Smoking, Raised blood pressure, Population over 65 and Cardiovascular fatalities were chosen in accordance with what the literature presents as risk factors, namely: [1, 37, 38, 40]. The remaining features were chosen to try to incorporate societal dynamics that can impact healthcare services [41, 43, 44]. Variable $x_{5}$ was chosen given the large number of corruption scandals involving Brazilian politicians. As a consequence, there is a significant amount of mistrust among the Brazilian population regarding its politicians and their ability to effectively handle public-health crises. There is also the perceived notion that regulations are poorly enforced in Brazil (variable $x_{6}$), which is of significant importance in the context of a pandemic.

Since some countries that present a small number of confirmed COVID-19 cases often have distorted CFRs, the analysis has excluded countries whose number of COVID-19 cases is lower than 200. After this pruning procedure, 75 countries still remain (which does not take into account Brazilian data), resulting in the following matrix of input data $\boldsymbol{X} \in \mathbb{R}^{10 \times 75}$

2 $$ \boldsymbol{X} \triangleq \begin{bmatrix} \boldsymbol{x}(1) & \boldsymbol{x}(2) & \cdots & \boldsymbol{x}(75) \end{bmatrix} , $$

where $\boldsymbol{x}(k) \triangleq [ x_{1}(k) \ x_{2}(k) \ \cdots \ x_{8}(k) ] ^{T}$ contains the eight features of the *k*-th country (see Table 1).

**Figure 12:**
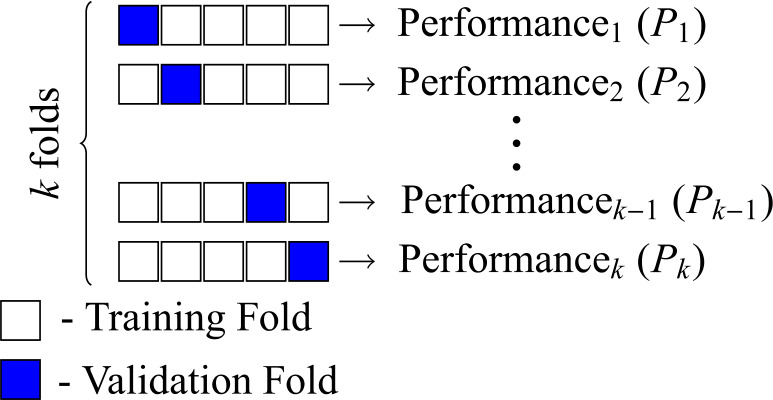
Ilustration of the *k*-fold procedure. In this paper, the performance is evaluated from the computation of the mean absolute error between the estimated CFR and the one computed from WOI data

Since the available data is unreliable, a careful data processing should be performed to guarantee a robust CFR prediction for the Brazilian case. The first processing procedure is executed to enhance the neural network accuracy (and to speed up training) by reducing the internal co-variate characteristics of the data [49]. In this first step, each entry of the matrix ***X*** is manipulated in order to obtain a normalised matrix $\tilde{\boldsymbol{X}}$, whose elements are computed as

3 $$ \tilde{x}_{i,j} = \frac{x_{i,j}-\hat{\mu }_{i}}{\hat{\sigma }_{i}}, $$

where $\hat{\mu }_{i}$ (resp. $\hat{\sigma }_{i}$) is the average (resp. standard deviation) of the *i*-th row of ***X***. The chosen neural regressor is the logistic feed-forward neuron, whose output, for a set of adjustable parameters $w_{i}$, $\forall i \in \{0,1,\ldots,m\}$, is described as

4 $$ y_{m}(j) = \frac{1}{1+\exp [-w_{0}-\sum_{i=1}^{m}w_{i}\hat{x}_{i,j} ]}, $$

where $\hat{x}_{i,j}$ is distinct from $\tilde{x}_{i,j}$ because of the feature extraction procedure. For a specific number *m* of principal components, there exists the respective neural estimate $y_{m}(j)$. Note that the neural regressor presents $m+1$ weights, since it also has a bias parameter. Because the neural architecture contains only one neuron, it is mathematically equivalent to the standard logistic regression. Feature extraction is an advisable step due to the insufficient number of training samples to enforce proper constraints in the neural network parameters; the desired estimation is considered a mathematical ill-posed problem [50]. This implies that over-fitting issues should be mitigated. One tool used for this purpose is Principal Component Analysis (PCA), which aims to obtain the most compact representation of a high-dimensional dataset in terms of the least square reconstruction error [51]. Loosely speaking, it can be described as an unsupervised linear dimensionality reduction technique that presents robust feature extraction properties [51]. The number *m* of principal components was selected by *k*-fold cross validation (a kind of model selection technique), in which the data set instances are randomly divided into *k* disjoint folds with approximately equal size, and every fold is in turn used to test the model trained from the remaining $k-1$ folds [52], as depicted in Fig. 12. The proposed committee regressor utilises the three neural regressors (each with a specific number *m* of principal components) that obtain the better performance in the set of 75 countries (which does not include Brazilian data).

Using $k=10$ folds and training the neural networks with the backpropagation algorithm under the mean quadratic error cost function, the mean absolute error (MAE) for each number of principal components is presented in Table 2. The MAE represents the mean absolute error computed by the differences between current CFR data for each country (obtained from WOI^1^) and the CFR estimated by a regressor. Algorithm 1 presents a pseudocode of the procedure that evaluates the MAE for a specific number *m* of principal components and assuming *k* folds in the *k*-fold cross validation.

**Algorithm 1:**
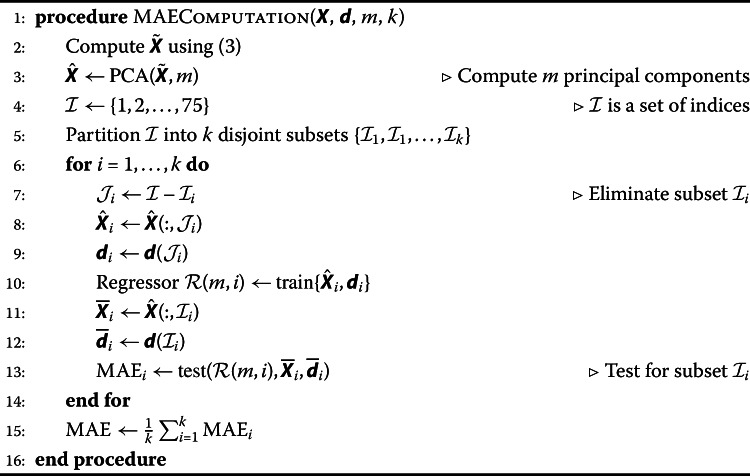
Evaluating MAE

**Table 2:** Computed MAE (using the *k*-fold strategy with $k=10$) for different numbers *m* of principal components. In bold: the three values of *m* that obtained better performance

*m*	MAE
**1**	**0.0187**
**2**	**0.0188**
**3**	**0.0189**
4	0.0192
5	0.0194
6	0.0194
7	0.0196
8	0.0199

Observe that architectures with one to three principal components perform better. These are selected in our study and provide the estimates in Table 3. Observe that the point-wise estimate (*i.e.*, obtained from the median of the estimates) of the neural committee for the Brazilian CFR is $\overline{\mathrm{CFR}} = 0.029$. Due to data inaccuracies and to the large differences in the estimated losses in human lives, the variability of such an estimate should be taken into account. In this context, it is more appropriate to adopt a prediction interval, which depends on the variability of the estimator. Since such a variability can be estimated by the *k*-fold cross-validation, one may compute the upper bound $\mathrm{CFR}_{\mathrm{up}}^{\alpha }$ of a confidence interval of *α*% [53]. Such an upper bound is $\mathrm{CFR}_{\mathrm{up}}^{68.27}=0.0545$ (resp. $\mathrm{CFR}_{\mathrm{up}}^{95}=0.0799$) for a confidence interval of 68.27% (resp. 95%). The median prediction is in line with the official statistics as of April 14 2020 (5.7%—www.worldometers.info). This suggests that either (i) the underreporting in death cases is similar to the underreporting in the overall cases, or (ii) the testing and reporting biases are captured by the selected variables in the model.

**Table 3:** The estimated Brazilian CFR with respect to the number of principal components. The median estimated is presented in boldface

*m*	Estimated CFR
1	0.0286
2	0.0299
3	**0.0290**

The weights with respect to each feature of the first principal components are described in Table 4. Such a table shows that variables $x_{3}$, $x_{4}$ and $x_{6}$ (*i.e.*, healthcare coverage, raised blood pressure and enforcement of regulations) have more relevance in the first three principal components, which indicates that they incorporate important information about the profile of each country. The eigenvalues of the PCA decomposition are shown in Table 5, which reveals the energy concentration in the first three principal components.

**Table 4:** Weight of each input variable in the first four principal components. The three most relevant weights of each principal component are emphasized in bold

Variable	$\mathrm{PC} _{1}$	$\mathrm{PC} _{2}$	$\mathrm{PC} _{3}$	$\mathrm{PC} _{4}$
$x_{1}$	0.0108	−0.0562	−0.1899	−0.2419
$x_{2}$	−0.0066	**−0.4152**	0.2447	−0.2238
$x_{3}$	**0.8342**	0.0150	**−0.3281**	−0.1498
$x_{4}$	**0.5694**	0.0761	**0.6638**	0.4199
$x_{5}$	−0.1176	**1.1189**	0.1200	**−0.4809**
$x_{6}$	**0.2586**	0.2200	**0.9944**	**−0.4558**
$x_{7}$	0.0665	**0.2309**	−0.1545	**1.3105**
$x_{8}$	0.1264	−0.1882	0.1256	0.0881

**Table 5:** Eigenvalues of the PCA decomposition. The first three principal components are able to retain 76.62% of the input features energy

Index	Eigenvalue
1	3.0100
2	1.9256
3	1.1121
4	0.5845
5	0.4940
6	0.3882
7	0.2349
8	0.1441

## Conclusions

Given the wide assortment of afflictions currently plaguing public available data over COVID-19, it is a challenging task to make reliable predictions concerning the spread and lethality of COVID-19. Consequently, data may be inaccurate and must be utilized with caution, which restricts the reliability of forecasting models constructed with them. It was already demonstrated that an inaccurate confirmed-case data induces nonidentifiability in the model calibrations, which helps to explain the wide range of forecasting variations [54]. For example, underreporting mild cases implies a reduction on the mortality rate [42, 55]. Unfortunately, such inconsistencies in reporting COVID-19 cases are a serious problem, which might sabotage the mitigation of its harmful effects and complicate the outbreak response [56]. Additional uncertainties derive from the fact that key characteristics of the transmissibility of COVID-19 (such as whether its transmission can occur before symptom onset) are currently unknown [57].

Yet, despite the apparent gaps in knowledge, it is still possible to gain invaluable insight. Namely, by combining the existing SEIR model with on/off lock-down policies one can see that the impact of the virus will be spread through multiple waves of decreasing amplitude. The results suggest that this trend persists even if the lock-down release is gradual. Such a scenario would effectively mean that there would exist multiple waves requiring flattening over time, in the absence of effective medication, an appropriate vaccine or the development of herd immunity.

Current epidemiological models such as SEIR are relatively simple, but robust prediction is dependent on reliable data. As a result, we developed a neural regressor that considers features that the current literature also deems as important factors in the fatality rate of COVID-19. This allows for non-linear extrapolations. Again, the issue of data unreliability surfaces. Through careful data processing alongside PCA and k-fold cross validation we believe that it is possible to obtain a more robust CFR prediction for Brazil, and possibly for countries with similar characteristics.

## Summary points

The following is a set of highlights of our paper:

- COVID-19 analysis focused on Brazil;
- COVID-19 SEIR Model with on / off lockdown strategy analysis
- Neural regressor with features that clinical studies present strong corroboration with COVID-19 fatality rate.

## Supplementary Information

Below is the link to the electronic supplementary material.

Supplementary information (ZIP 4 kB)

## Data Availability

All data employed was obtained from the herein cited public sources. The R code developed for simulation was annexed to this work as Additional file 1.

## Footnotes

1 From www.worldometers.info.

## Abbreviations

BCG: Bacillus Calmette-Guérin
CFR: Case Fatality Rate
ILO: International Labour Organization
LT: Legatum Institute
MAE: Mean absolute error
PCA: Principal Component Analysis
SEIR: Susceptible, Exposed, Infected, Removed
SIR: Susceptible, Infected, Recovered
WBDI: World Bank Development Indicators
WEF: World Economic Forum
WJP: World Justice Project
WOI: worldometers.info
WHO: World Health Organization
